# The case for orthopaedic medicine in Israel

**DOI:** 10.1186/2045-4015-2-42

**Published:** 2013-11-18

**Authors:** Aharon S Finestone, Simon Vulfsons, Charles Milgrom, Amnon Lahad, Shlomo Moshe, Gabriel Agar, Dan Greenberg

**Affiliations:** 1 Department of Orthopaedics, Assaf Harofeh Medical Center, Zerifin, Israel; 2 Sackler Faculty of Medicine, Tel Aviv University, Ramat Aviv, Israel; 3 Israel Society of Musculoskeletal Medicine, Israel Medical Association, Ramat Gan, Israel; 4 Department of Health Systems Management, Ben-Gurion University of the Negev, Beer Sheba, Israel; 5 Institute of Pain Medicine, Rambam Health Care Campus and Rappaport School of Medicine, Technion, Haifa, Israel; 6 Department of Orthopaedics, Hadassah University Hospital, Jerusalem, Israel; 7 Department of Family Medicine, Hebrew University of Jerusalem Medical School, Jerusalem, Israel; 8 Maccabi Healthcare Services, Occupational Medicine Department, Holon, Israel

**Keywords:** Orthopaedic medicine, Musculoskeletal pain, Pain management

## Abstract

**Background:**

Musculoskeletal complaints are probably the most frequent reasons for visiting a doctor. They comprise more than a quarter of the complaints to primary practitioners and are also the most common reason for referral to secondary or tertiary medicine. The clinicians most frequently consulted on musculoskeletal problems, and probably perceived to know most on the topic are orthopaedic surgeons. But in Israel, there is significant ambivalence with various aspects of the consultations provided by orthopaedic surgeons, both among the public and among various groups of clinicians, particularly family practitioners and physiotherapists.

**Methods:**

In order to understand this problem we integrate new data we have collected with previously published data. New data include the rates of visits to orthopaedic surgeons per annum in one of Israel’s large non-profit HMO’s, and the domains of the visits to an orthopaedic surgeon.

**Results:**

Orthopaedic surgeons are the third most frequently contracted secondary specialists in one of the Israeli HMO’s. Between 2009 and 2012 there was a 1.7% increase in visits to orthopaedists per annum (P < 0.0001, after correction for population growth). Almost 80% of the domains of the problems presented to an orthopaedic surgeon were in fields orthopaedic surgeons have limited formal training.

**Discussion:**

While orthopaedic surgeons are clearly the authority on surgical problems of the musculoskeletal system, most musculoskeletal problems are not surgical, and the orthopaedic surgeon often lacks training in these areas which might be termed orthopaedic medicine. Furthermore, in Israel and in many other developed countries there is no accessible medical specialty that studies these problems, trains medical students in the subject and focuses on treating these problems. The neglect of this area which can be called the “Orthopaedic Medicine Lacuna” is responsible for inadequate treatment of non-surgical problems of the musculoskeletal system with immense financial implications. We present a preliminary probe into possible solutions which could be relevant to many developed countries.

## Background

The public demand in Israel for being examined by orthopaedists seems to be rising. The HMO’s generally respond to this demand by increasing the supply. This increase in service is responsible for direct and indirect increases in expenditure, not necessarily related to any increase in measurable health parameters and is contrary to all official and unofficial recommendations on how the Israeli medical system should be managed [1]. There is concern among primary physicians and other clinicians with the effectivity of the service provided by orthopaedists in both public and private clinics. Even though about a quarter of primary physicians' visits are in the general field commonly referred to as orthopaedics, and visits to orthopaedists are among the most common types of specialist visits^a^, very little analysis of the services rendered in this field has been reported.

While there is a considerable debate in the medical literature concerning justification for orthopaedic surgery indications (especially spine surgery), surgery is a relatively rare endpoint for an outpatient orthopaedic encounter in Israel. Therefore surgery is not necessarily the largest item in the public expenditure related to orthopaedics. The weight of orthopaedics in the community medical services may be seen by the number of independent^b^ orthopaedists contracted by the Maccabi Healthcare Services^c^ (Table 1) exceeded only by a few other medical disciplines^d^.

**Table 1:** Independent physicians in the Maccabi HMO, Atzmaiton, January 2013

**GP, family & internal**	**755**	**26.0%**
Pediatrics	436	15.0%
Obstetrics & gynecology	330	11.4%
Ophthalmology	219	7.5%
Orthopaedics	205	7.1%
Surgery	197	6.8%
ENT	162	5.6%
Dermatology	154	5.3%
Psychiatry	104	3.6%
Gastroenterology	73	2.5%
Urology	68	2.3%
Neurology	68	2.3%
Cardiology	52	1.8%
Pulmonology	19	0.7%
Endocrinology	17	0.6%
Allergy	16	0.6%
Haematology	10	0.3%
Nephrology	9	0.3%
Rheumatology	9	0.3%
Oral medicine	3	0.1%
Pain	1	0.0%
Total	2907	100.0%

The purpose of this integrative study is to create a preliminary analysis of how the orthopaedic system works in Israel, leading to a preliminary discussion of what changes can improve the delivery of orthopaedic care.

### The clinical scope of orthopaedics

Orthopaedics classically includes the diagnosis and treatment of ailments of bones, joints, muscles, tendons and ligaments. This includes various types of illnesses and injuries, a variety of exercise and non exercise related musculoskeletal complaints, trauma and age related changes. Trauma of the musculoskeletal system includes relatively minor events such as simple ankle sprains and other ligamentous injuries, dislocations, joint instability, contusions, lacerations, while major trauma includes fractures and penetrating injuries. Two other fields in orthopaedics, most frequently associated with senior citizens are degenerative joint disease and osteoporosis.

Of patients visiting primary physicians (general practitioners, and family specialists), 14-28% of the complaints refer to the musculoskeletal system [2-4]. Among patients between the ages of 36 and 49 years old, about 40% of the complaints are related to musculoskeletal pain [5]. Musculoskeletal pain & trauma comprise the most common reason for referral to secondary or tertiary medicine [6].

There is a large range of clinicians involved in treating diseases of the musculoskeletal system. General practitioners, family medicine specialists and orthopaedic surgeons are probably the most frequently consulted medical doctors for musculoskeletal problems. Other medical (MD) specialists include sports physicians, internists, rheumatologists, physiatrists, rehabilitation specialists & neurologists. Non MD clinicians that treat musculoskeletal pain include physiotherapists, chiropractors and osteopaths. Utilization of complementary and alternative medicine (CAM) is also continually increasing [7].

### The orthopaedic surgeon

**Figure 1:**
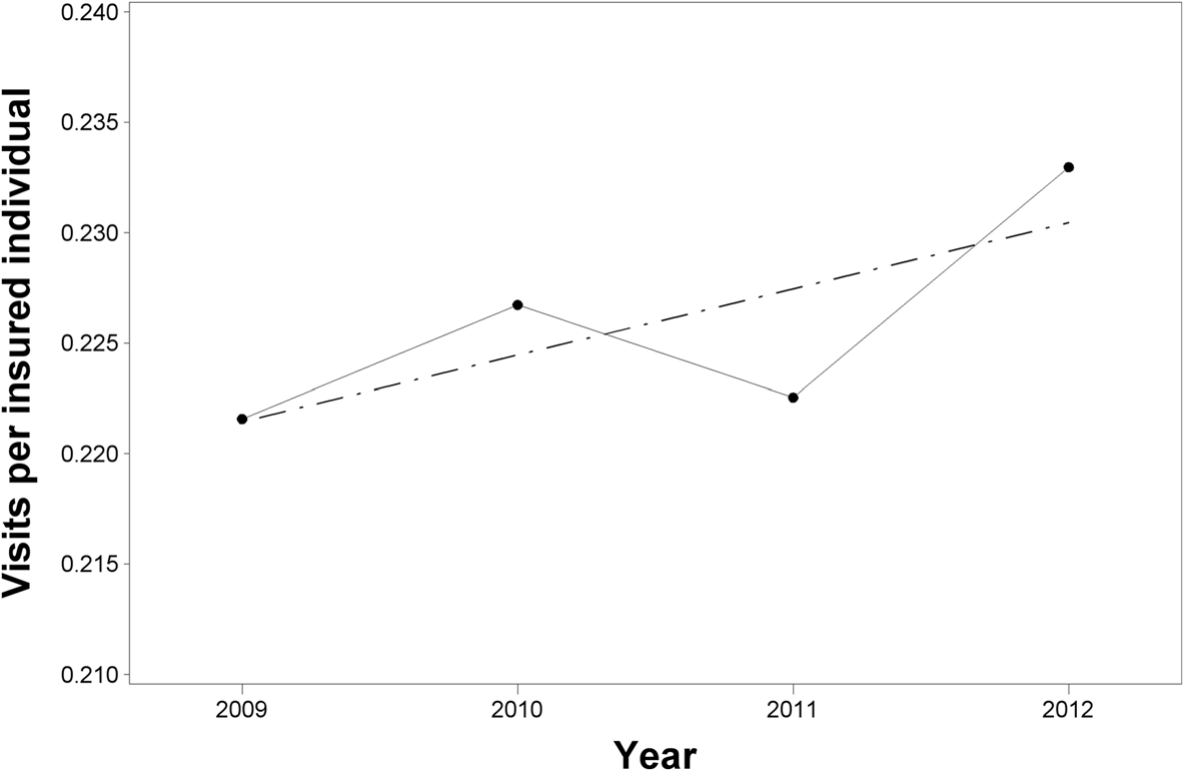
**Annual trend in orthopaedic visits per insured individual (at beginning of each year) at one HMO.** Annual data for 2009–2011, data of 2012 extrapolated from first 10 months. There is an annual increase of 1.7% (P < 0.0001 logistic regression, SAS). For 2011, these represent 2.8% of all medical encounters in the HMO. This ratio increased to 3.0% in 2012.

Orthopaedics has evolved to be primarily a surgical discipline. In Israel, orthopaedic surgeons are probably perceived by the public as having the most knowledge on the topic of managing problems of the musculoskeletal system. A result is the high demand for orthopedists in Israel, particularly since the necessity for a referral from a primary physician was cancelled in 1993 [8]. The orthopaedic utilization rate in one of the Israeli HMO’s seems to be increasing annually by one percent (Figure 1).

### The orthopaedic residency

The surgical demands on the orthopaedic surgeon are increasing at a tremendous rate. New information leading to improved understanding of diseases and the evolution of new technologies and operating techniques all make the training of the next generation of orthopaedic surgeons a challenge to any chairman running a residency program. It is now becoming hard to cover all surgical knowledge in a six year orthopaedic residency. A quarter century ago, an orthopaedic surgeon would have been expected to be proficient in some eight textbook volumes. Nowadays, just to be a specialist in any orthopaedic subspecialty (e.g. foot and ankle surgery) requires proficiency in eight volumes in that subspecialty. As an immediate consequence of this^e^ the orthopaedic residency program in Israel has been forced, over the recent years, to gradually drop everything not immediately related to orthopaedic surgery, including most of the rotations outside the orthopaedic department. The likelihood of a resident doing a rotation such as in rehabilitation (formally called physical medicine and rehabilitation, where he might be trained on what happens to his patients after surgery) is very low. Even orthopaedic pathology has been removed from the curricula for specialists' exams in Israel. Most orthopaedists finishing residency 25 years ago were capable of handling most trauma cases as well as basic orthopaedic surgery. Because of the broadening of surgery and the increased complexity of surgical techniques, nearly all recently trained orthopaedist need to do extra training to become surgically proficient. The orthopaedic surgeon’s training is becoming much more technical and narrow, with much less emphasis on general orthopaedic surgery and orthopaedic medicine. A new trend is to let a resident decide on his/her field of subspecialty towards the end of the fourth year of the six year program, and let him/her devote a major part of the last two years of their residency to specializing in that field.

### Deficiencies in orthopaedic surgeon's knowledge

**Figure 2:**
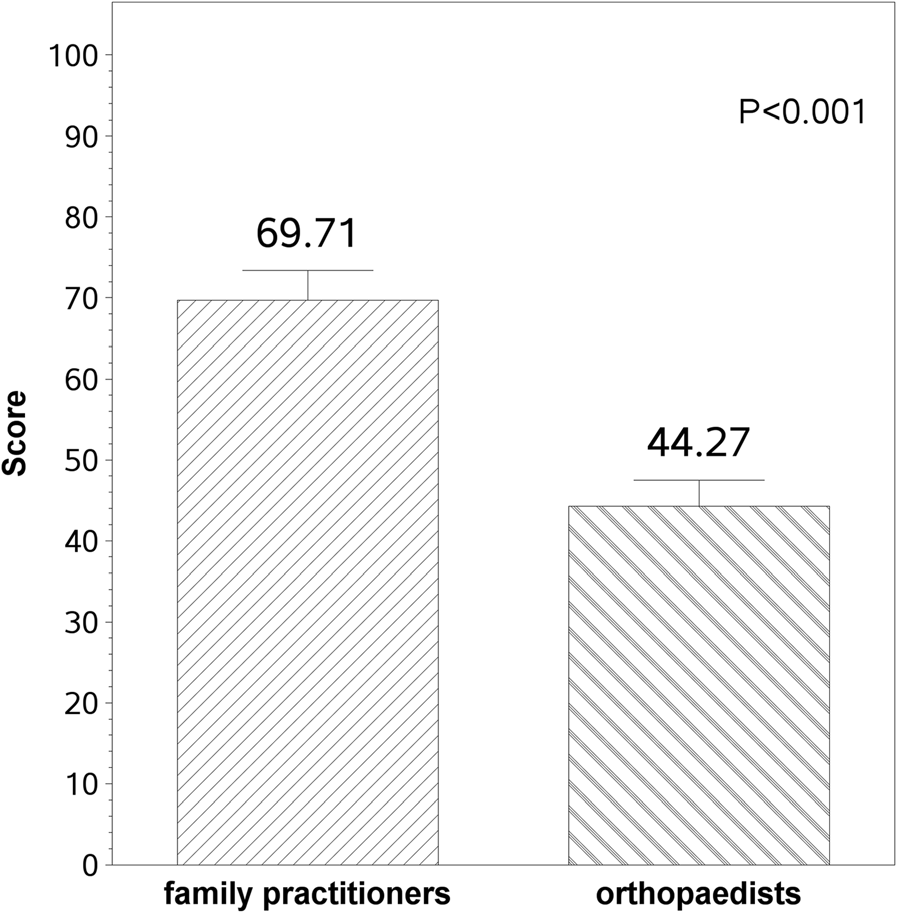
**Family practitioners and orthopaedists knowledge on the treatment of simple low back pain as assessed in a questionnaire based on the Israeli guidelines for treatment of LBP **[9]**.**

While orthopaedic surgeons are obviously the senior authority on managing surgical cases in their field, their training in non-surgical orthopaedics is often deficient. This lack of knowledge is probably only one of the contributing factors to dissatisfaction of many clinicians (e.g. primary physicians and physiotherapists) with the treatment provided by orthopaedic surgeons dealing with non-surgical issues. Amongst other things, this results directly from the lack of formal education on non surgical management of complaints of the musculoskeletal system. Based on the proliferation of data the orthopaedic surgeon has to master on surgical techniques, this is not likely to change. While the resident in orthopaedic surgery does do some supervised clinic work as part of the residency, its primary focus is in selecting those patients likely to benefit from surgery. This approach often leads the orthopaedist to have a high index of suspicion that the patient needs surgery. The orthopaedics surgeon’s clinical algorithms are based on this high index of suspicion. It made some sense when the patient did not have direct access to the orthopaedist, and was referred by a gate keeper who only referred if he thought the patient needed surgery (a system that existed in Israel before 1993). The needs of the public are often not well served if they approach the orthopaedic surgeon directly, as orthopedic surgeons are not proficient in diagnosing or treating non-surgical issues. One of the fields illustrating this problem is the treatment of simple low back pain, one of the most common problems orthopaedists encounter (Figure 2) [9]. Knowledge of treatment management guidelines for lower back pain among orthopaedists is less than that of family practitioners in Israel, yet patients seek orthopaedists for their back pain care. Until recently, orthopaedic surgery textbooks have looked upon low back pain as a surgical entity, needing imaging evaluation with myelography, CT or MRI [10,11]. Guidelines for managing low back pain had already been published in many countries, with one of their intentions being to limit referral for imaging.

Orthopaedic surgeons are not well trained in patient communication techniques, which are important in managing musculoskeletal complaints and particularly backache [12]. Most have never heard of concepts such as neuro-linguistic programming^f^ and are not aware that with most musculoskeletal complaints, the physician's manner and terminology are more important to the recovery of the patient than what imaging is performed. It is not known whether these deficiencies are related to correctable problems in the orthopaedic residency. Orthopaedics is known to be somewhat extra-territorial to medicine. This is, to a major extent, because the milieu is so different from medicine (hammers, power drills, saws, screws etc.). A direct consequence of this is the type of personality orthopaedics attracts, mainly someone who wants to fix the problem and move on, and less interested in many fields most physicians are.

### Deficiencies in general practitioner’s training in musculoskeletal medicine

In 1993 direct access to orthopaedists was allowed without primary examination of family doctors in Israel, primarily in the Clalit HMO, and shortly followed by all others^g^. One might argue that opening direct access was a mistake, and that it is better when orthopaedic patients first see their family specialist, and are diagnosed, treated or referred at his/her discretion, as the orthopaedic surgeon does not, as a rule, take the patients' general health into account when prescribing potentially dangerous drugs such as NSAID's. But data from Israel and abroad suggest general practitioners are not well enough equipped to do this pre-selection [4,13]. Their training in musculoskeletal medicine in medical schools is lacking. Not all medical schools in Israel teach the anatomy of the musculoskeletal system. It is only recently that medical students in some of the faculties have been exposed to pain clinics. In the two weeks (7–9 days) that students spend in orthopaedic surgery, there is an overwhelming amount of material to learn on managing surgical problems (e.g. septic arthritis, musculoskeletal trauma). There is little or no time devoted to non-surgical problems.

The fact that there is a deficiency in teaching musculoskeletal disease in medical school is well known. Ahern et al. report that significant musculoskeletal disease in patients admitted to general medical wards in Australia is often inadequately assessed or even ignored [14]. Akesson et al. reported on this in the WHO bulletin on “The Bone and Joint Decade” showing that while musculoskeletal disease comprises 14-28% of patients complaints in Canada, the topic is only allocated 2.3% of curriculum hours, and is compulsory in only 12% of the programs [4]. They also note that in the 4–6 week orthopaedic clerkship in UK medical schools, 88% of the time is for teaching hospital oriented musculoskeletal problems requiring surgery. A further point they make is that there is also a lack of appreciation of the importance of psychological factors in chronic musculoskeletal disease [15]. In a focus group survey of GP’s, rheumatologists, orthopaedic surgeons and geriatricians in the UK, Coady et al. found lack of agreement on what needs to be taught, lack of confidence in teaching amongst non-musculoskeletal specialties, and poor communication between specialties [16].

In 1998, Freedman and Bernstein developed a musculoskeletal examination to test health-care providers with respect to their basic cognitive understanding of musculoskeletal problems [17]. This examination has been used to compare the knowledge of different groups of clinicians at different stages of training [18-20]. On these assessments physiotherapists score better than all groups of physicians, excluding orthopaedic residents [20]. But reviewing the questions composing the test shows that 24 of the 25 are on standard knowledge that is covered in orthopaedic surgery (including trauma), and not related to non-surgical management. Only one question is in the realms of orthopaedic medicine (the question on the muscles involved in "tennis elbow"). Even that question is not related to the myofascial explanation of how pain in the extensor muscles of the wrist can be referred to the lateral epicondyle.

In Israel, Mashov et al. also found a severe lack of knowledge of orthopaedic medicine. They have initiated several post graduate programs improving primary physicians’ management of these problems, and they also support the recommendation of Akesson et al. to lengthen the orthopaedic clerkship of medical students to at least 6 weeks [4,13]. It is not surprising, therefore, that some GP’s are too willing to delegate responsibility to the orthopaedic surgeon rather than enter a possible confrontation with the patient in an area where they may know less or feel less confident.

### The "orthopaedic medicine lacuna"

Our main claim in this paper is that there is a neglected field in medicine, orthopaedic medicine. There are no departments of orthopaedic medicine paralleling departments of orthopaedic surgery (as exist for neurosurgery, cardiac surgery, plastic surgery, etc.). The consequence is a lack of knowledge in this field by all involved. The fact that there is no orthopaedic medicine department means that medical students don’t rotate there and do not study orthopaedic medicine (as they do orthopaedic surgery). The fact that there is no department also means that there is no chairman pushing his subordinates to do research in the field. Even the leading journals in orthopaedic surgery (e.g. The Journal of Bone and Joint Surgery^h^) are less than eager to publish papers on non-surgical topics. The first step to understand this lacuna is to define the scope of orthopaedic medicine and see how training can be accomplished in this field.

### The orthopaedic clinic: the clinical spectrum vs. the orthopaedic surgeon's training

A fact obvious to all (patients and clinicians) is that most patients attending most general orthopaedic clinics do not need surgery. That being the case, it is necessary to analyze what type of problems bring patients to the orthopedic clinic. We are not aware of any studies reporting this, so we created a list of clinical problems likely to be presented by patients approaching an orthopaedic surgeon (Table 2, column 2) and categorized them into domains of interest or training (column 3). While the first five lines are clearly part of an orthopaedic surgeons' training in Israel, the rest are not^i^.

**Table 2:** Common reasons for visiting a general orthopaedic clinic (whether self referral or referral by another clinician) and relevant domains of knowledge

	**Visit reasons**		**Relevant domain of training**
1	Minor trauma		Orthopaedic trauma / general trauma
2	Major orthopaedic trauma		Orthopaedic trauma / general trauma
3	Orthopaedic surgery including scoliosis and pediatric orthopaedic surgery		Orthopaedic surgery
4	Tumors of the musculoskeletal system		Orthopaedic surgery
5	Osteoarthritis	Needing surgery	Orthopaedic surgery
6		Not needing or S/P surgery	Rehabilitation
7	Follow up of fractures		
8	Rehabilitation		Rehabilitation
9	Osteoporosis		Family medicine / endocrinology
10	Musculoskeletal pain		Localized muscle pain
11			Radiated muscle pain
12			Pain management
13	Orthopaedic appliances (orthotics, braces and shoes)		Orthotics & prosthetics
14	Orthopaedic furniture (chairs, beds and mattresses)		Ergonomics
15	Complaints related to exercise / sports		Exercise medicine^o^
16	Sick leave, temporary and permanent work restrictions		Occupational medicine
17	Somatization		Family medicine / clinical psychology
18	Litigation issues		Legal medicine

**Figure 3:**
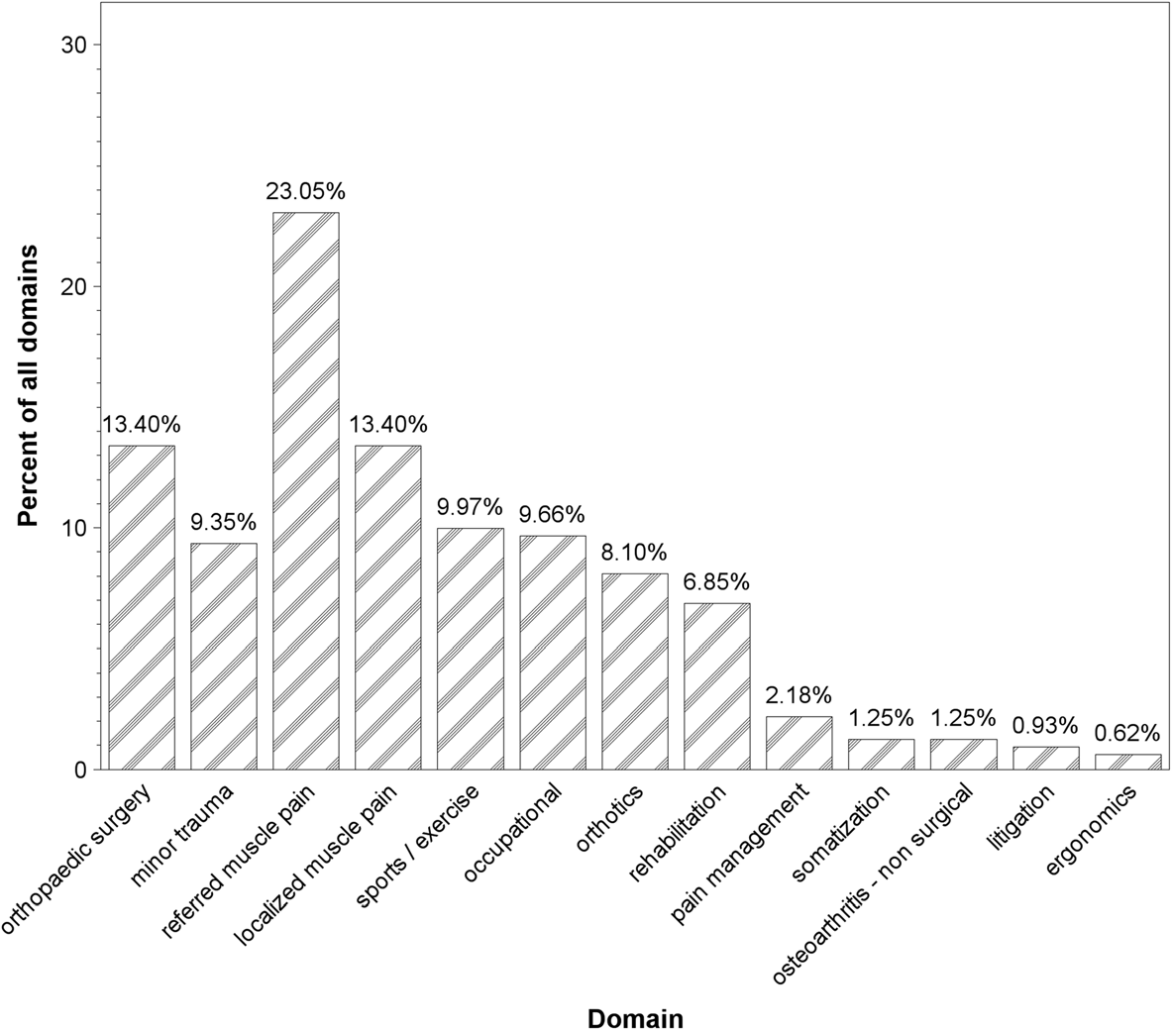
**Common reasons for visiting a general orthopaedic clinic (whether self referral or referral by another clinician) and relevant domains of knowledge.** 52% were seen in a standard HMO orthopaedic clinic, 30% in a military orthopaedic clinic, and 18% in a hospital outpatient clinic. There were slightly more orthopaedic surgery domains in the outpatient clinic, slightly more occupational domains in the military clinic, and slightly more sports and localized and referred muscle pain in the HMO clinic.

We further prospectively categorized 215 consecutive patients (8 clinic shifts) examined by one of us (ASF) into one or two of these domains, at the end of each patient encounter. Data were collected in 2012 in an urban setting. In all, there were 321 domains (1.5 per patient). The domains are presented in Figure 3, after grouping all orthopaedic surgery (items 1–5 in Table 2) into one heading.

Reviewing the orthopaedic surgeons 6 year residency training programs shows that most of them have less than an hour of formal training in each of these topics. One might argue that the orthopedic surgeon is also a qualified MD, and will have learned the approach of a general physician to common problems of the musculoskeletal system. However, as we stated previously, the training in medical school does not give sufficient training in managing musculoskeletal problems, be they surgical or not. Another problem is that material covered in medical school and not reinforced in residency is not likely to have a great impact on a specialist's clinical behavior.

### The case of orthotics

The case of orthotics illustrates the fallacy of orthopaedic medical care given by orthopaedic surgeons. The Israeli HMO's and insurance companies frequently insist on a referral from an orthopaedic surgeon in order to dispense an orthotic (limiting family practitioners prescribing simple aids, supposedly as a cost saving measure). These include foot orthoses and any other orthopaedic appliances. This is in spite of the fact most orthopaedic surgeons know very little about orthotics since the orthopaedic residency does not include even one compulsory hour on the subject. The annual expenditure in Israel on shoe orthotics alone is estimated at NIS 100M (Table 3). These data are consistent with the large number of people employed in the field in Israel (several hundred employees, manufacturing expenses and importation of many of the orthotics including custom made shoe orthotics manufactured by CAD-CAM technology costing NIS 2,400). Other more expensive devices such as braces for scoliosis and knee braces for sports injuries can cost up to 10,000 NIS each. There is very little scientific knowledge in the field, and almost no quality assurance. More troublesome are the relationships that sometimes exist between the prescribing clinicians and the manufacturers or agents. In the pharmaceutical world, there are clear regulations about conflict of interest. The world of orthotics is simply extra-territorial, with no clinical field involved in its management. In these times of financial constrain, it is necessary to analyze whether this “health care” expenditure, together with the additional expense on the associated orthopaedist referrals, provide a discernible benefit on any public health parameter in Israel.

**Table 3:** estimated expenditure on custom made shoe orthotics

**HMO**	**No. prescriptions**	**Price paid per prescription**	**Total**
HMO-a	30,000	600 NIS	18M NIS
Other HMO's	60,000	800 NIS	48M NIS
Israel defense forces	10,000	200 NIS	2M NIS
Private market	20,000	1,000-2,400	32M NIS
Total	120,000		100M NIS

Unofficial data from 2010. Price for HMO-a includes the price paid by the HMO, the deductable, and an estimate of the surcharge paid to improve the orthotics from the basic covered by the HMO. Other HMO's were calculated counting 25% of citizens in HMO-a, and 2/3 rate of utilization per person compared with HMO-a. The price for HMO-a is lower than other HMO's because they contract the orthoticians directly. The data in this table is provided as an overview and constitutes a rough estimate. Exchange rate: $1 = 4 NIS.

### The case of myofascial pain

Myofascial pain comprises a common syndrome of localized or referred muscle pain ensuing from a trigger point in a muscle. It is a leading cause of muscular disability of the shoulder girdle, neck, and lumbar regions [21], and is the reason for many common episodes of prolonged inability to work. As demonstrated in Figure 3, muscle pain can account for up to 40% of the orthopaedist’s encounters, but the subject is not taught in medical school or in orthopaedic residency. The anonymity of myofascial pain is a further consequence of the orthopaedic medicine lacuna. It is estimated that globally, 1 in 5 adults suffer from pain and another 1 in 10 adults are diagnosed with chronic pain each year [22]. Muscles play a major role in many pain syndromes sometimes as a direct cause of the pain, and at other times as a mediator. Even though muscle constitutes 40% of the body, it is the only organ not linked to a specific medical specialty [23]. It has been known that muscles cause pain, local and referred for many years. As far back as the 16th century, French physician Guillaume de Baillou (1538–1616) described what is now known as myofascial pain syndrome (MPS) [24]. In 1816, the British physician Balfour reported observing muscles with "nodular tumors and thickenings which were painful to the touch, and from which pains shot to neighboring parts" [25]. In modern medicine, Jonas Kellgren, the British rheumatologist famous for the staging of osteoarthritis, described localized and referred pain patterns caused by muscles and ameliorable to injection of local anesthetic into the muscle in the 1930's [26,27]. Another prominent British physician, James Cyriax initiated the field of orthopaedic medicine, developing various manual treatment techniques including deep friction, massage and manipulation [28]. Most of his teachings have not interested physicians, but have been implemented by physiotherapists in the conservative treatment of musculoskeletal pathology.

In the second half of the 20th century, Janet Travell and David Simons, physicians working in the USA, developed the field considerably, publishing their work in a detailed textbook [29]. In 1979, Lewit, A Czech physician reported that the analgesic effect was not dependent on the local anesthetic injected. This paved the way to "dry needling" treatment without medication, the response being related to the electric effect of the metal needle inserted into the muscle and the ensuing reflex [30]. This has been further developed using safer needles [31]. Physicians frequently tell patients with pain (dolor) that they have inflammation. This is in spite of the absence of rubor, tumor, calor and functio laesa, the classical clinical findings necessary to diagnose inflammation, and in the absence of histopathological signs of inflammation in series where biopsies were taken) [32]. This explanation to the patient may reflect physician's preference to prescribe NSAIDS rather than simple pain killers.

A recent review on knee pain in patients with osteoarthritis ignores the role of muscles and myofascial syndrome in mediating the pain [33]. The 13 year long training of orthopaedic surgeons beginning with medical school does not include even one hour on muscle pain. Their training on muscles is largely limited to surgical anatomy.

### The case of imaging

It is well known that in certain settings, orthopaedic surgeons will not see a patient prior to imaging, be it an X-ray, a CT scan or an MRI [34]. This approach may well be justified if the patient is referred by a gate keeper who thinks the patient needs surgery. It would be irresponsible to operate on a patient's back without some form of 3D imaging, and most authorities agree that a knee MRI should be performed before an arthroscopy. But some of these imaging modalities are costly, and are therefore restricted by HMO's and insurers. As a consequence, orthopaedic surgeons, GP's and the public are trained to think that the most important way to diagnose musculoskeletal problems is imaging, and physicians seem to be forgetting how to perform a physical examination [34,35]. This causes increasing pressure to do more imaging. So much so that imaging has even been coined "idolatry" [36]. Frequently, patients think radiography will tell them the source of the problem [12]. This is true in only a small fraction of the cases. Some primary physicians know the limited value of imaging [9,12] and they restrict its use. So a possible reason for a patient going directly to an orthopaedic surgeon might be to be referred for imaging that the patient desires. Pham et al. recently reported that 28.8% of elderly patients with acute low back pain underwent imaging within 28 days and an additional 4.6% between 28 and 180 days. Among patients who received imaging, 88.2% had simple radiography, while 11.8% had a CT or an MRI as their initial study [37]. This is clearly in violation of most guidelines for the treatment of acute low back pain that recommend not doing any kind of imaging earlier than 6 weeks. Rolfe et al. estimated that approximately one in three imaging tests in the United States are performed in situations in which clinical benefit is unlikely to outweigh risks, yet few clinicians discuss these risks with patients undergoing tests, and even when they do, patients' knowledge about the risks does not change their decisions substantially [38].

**Figure 4:**
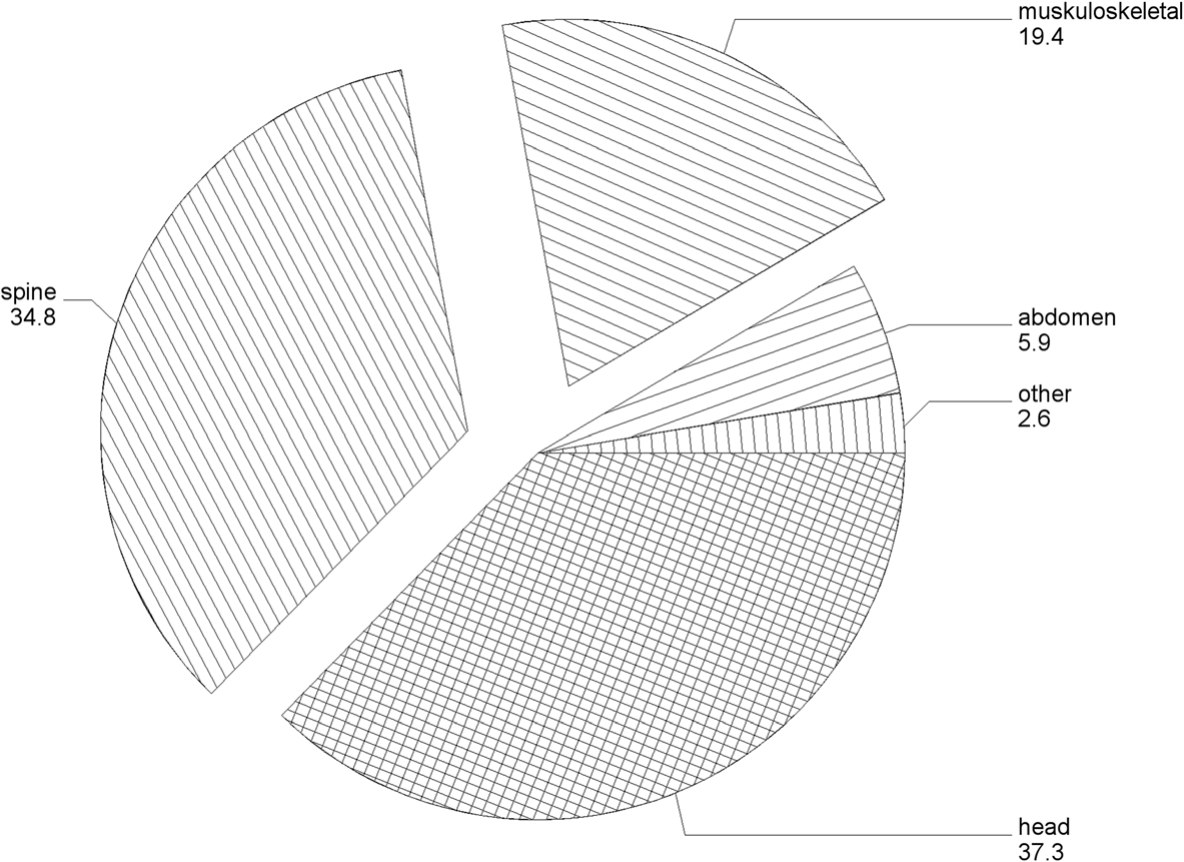
**Percentage of 6.4 million MRI's performed by body area, 2010, Medicare USA, adapted from Sharpe et al. **[40]**.**

A major component of the expense of imaging is now MRI. Many of these are for musculoskeletal imaging. More than half the MRI's performed in Israel in the late 1990's were of the musculoskeletal system [39] and newer data from the USA are similar (Figure 4) [40]. A relatively mild system of preauthorization of imaging requests found that 23% & 15% of musculoskeletal and neuro-radiology tests ordered were deferred [41].

Imaging has several drawbacks. One is the anxiety that ordering imaging causes the patients, who frequently do not understand the purpose of the test, but are sure the clinician suspects something at least as serious as cancer. While a common claim is that a normal imaging test will alleviate the patient’s anxiety, a recent meta-analysis found no benefits of diagnostic testing on reducing illness worry or anxiety, although only a few studies examined these outcomes. Moreover, no effect on symptom status was found [38]. There was also a trend for better outcomes in backache without “red flags”^k^ when imaging is not performed [12,42]. Another factor against imaging is the fact that most people over the age of 30 start developing degenerative changes and these are wrongly perceived by the patient to indicate illness, warranting further testing and even surgery. This is so much so that Roland et al. proposed that radiologists changed the way they report [43].

Some imaging modalities carry the dangers of ionizing radiation. It is estimated that 1.5-2% of malignancies in the USA are attributable to CT scans alone [44]. In Israel 1% of the population perform a bone scan every year [45,46], calculated to cause a radiation induced fatality rate of 1/4,000 scans^l^, some 15 cases per annum, with another 30 non-fatal malignancies. There is little doubt that the vast majority of these bone scans does not contribute positively to the management of the patients.

A further drawback is the vast expense to the healthcare budget. This is not only related to the primary imaging, but also to tests required because of false positive tests. The rate of detecting a serious condition with imaging may be as low as 0.5% to 3.0% when diagnostic tests are ordered in patients with a low probability of disease. This means that a diagnostic test with a 90% sensitivity and 90% specificity would yield 4 to 19 false-positive results for every true-positive result in patients for whom the test is ordered simply to rule out a disease for which the clinical suspicion is already low [47]. The consequence is even more tests done, not for a clinical complaint, but for false positive results of previous unnecessary imaging.

This abuse of imaging and its consequences have two main causes. The first is clinicians' ignorance of musculoskeletal medicine. One of the more frequent reasons for ordering imaging is the clinicians' not having a good idea of what the patient’s diagnosis is. While it is obvious that imaging is supposed to provide information, it is not intended to replace a good clinical assessment. In order to use imaging efficiently, the clinician has to have made a clear differential diagnosis based on history, physical examination, and a clear knowledge of what he is looking for. As stated previously, when not used to answer a specific question, imaging will inevitably be inefficient. The second reason for imaging abuse is the absence of a discipline with a broad view of both musculoskeletal medicine, its epidemiology, and the administrative issues involved (e.g. expenditure on imaging). This discipline would be able to make clear statements on when imaging is likely to be necessary and when not, giving the tailwind to guidelines published by family practitioners. Orthopaedic surgeons, with their narrow mechanistic view on the musculoskeletal system have not done this, and cannot be expected to do so.

### Economic implications of the orthopaedic medicine lacuna

The national cost of pain in the USA is estimated to exceed $500 billion [48]. Half of this is thought to be from musculoskeletal pain, and half of that related to back pain. As previously stated, the highest authority on managing these patients, the orthopaedic surgeon is trained to diagnose problems amenable to surgery. These include fractures, tumors, osteoarthritis and herniated disks with progressive neurological deficit (diagnoses that are in the consensus). But in the management of the vast majority of these patients, the orthopaedist surgeon has no postgraduate training. Almost all diagnoses in orthopaedic surgery necessitate imaging for treatment. The orthopaedic surgeon is basically trained not to move without imaging. This attitude toward management of surgical cases may be justified, but is not the correct management of most non-surgical cases. This causes the service provided by orthopaedic surgeons to be expensive, due to its utilization of expensive technologies, both in the field of diagnostics (scans, CT, MRI) and in the field of treatment (injections, surgery) [49,50].

Treatment for spine related disorders for example, has become increasingly specialist-focused, imaging-oriented, invasive and expensive [51]. According to Deyo et al., between 1994 and 2004 Medicare expenditures in the US increased 629% for epidural steroid injections, 423% for opioid medications, 307% for MRI's and 220% for lumbar fusion surgeries [52]. With no clear cut evidence of improved population health resulting from these expenses, a major question is whether the health system is not simply passing the buck with patients with musculoskeletal disorders.

Given the very basic knowledge that primary practitioners have of musculoskeletal medicine, patients may justifiably want a specialist opinion on their condition. This is directly translated into going to see an orthopaedist (they may not know he is only trained in surgery), without realizing that in 80% of their ailments, the orthopaedic surgeon is no more competent than their family specialist. Our medical systems are not offering anything else. The orthopaedist, apart from ordering imaging, is also likely to refer the patient to an orthopaedic subspecialty. Neither can diagnose the patient's non-surgical complaint, and the patient can continue wandering between physicians, imaging tests and alternative medicine for months and even years, suffering, and wasting medical resources that don't help him/her. A recent study in Israel on ankylosing spondylitis demonstrated an average of almost 6 years delay in diagnosis [53].

Another issue with vast economical implications, but with next to no clear data, is related to orthopaedic surgeons' recommendations in the domain of occupational medicine. This includes recommendations for sick leave or limited duty following disease (e.g. backache) and permanent limitations [54].

### Research implications

In recent years, a considerable body of information has been developed on musculoskeletal medicine, some of which is evidence based. Organizations and disciplines supporting this are the physiotherapist organizations, the American Academy of Orthopedic Medicine and the Israeli Society for Musculoskeletal Medicine and its European counterparts. But a lot of the treatment, whether physical, or recommendations in the fields of sports or occupational medicine, lack the rigorous research background customary in modern medicine. There is a problem defining rigid outcome criteria that are not affected by other factors. No less important is the fact that most aspects of non-surgical musculoskeletal medicine do not need specific drugs or equipment, resulting in a lack of interest and funding from pharmaceutical and medical equipment companies.

Beyond the necessity to improve our scientific data on medical aspects of orthopaedic medicine is the need to identify and quantify the economic burden of orthopaedic problems in Israel and the patterns of the futile circles our patients are doing before getting diagnosed and treated. Following this, it makes sense to devise algorithms to limit these futile circles. Furthermore, the economical impact of many of the domains treated by orthopaedic surgeons with no training in that domain (such as orthotics and occupational medicine) should be investigated.

### Directions for improvement

While it is quite clear from all that has been stated so far that musculoskeletal pain and its management (or better stated, lack of management) pose a serious health policy problem, there is no simple solution. The current trend in medicine is fragmentation of disciplines into subspecialties, and there is a dire need for an opposite trend [1]. In the following section we present a preliminary review of some of the options that might be incorporated into a solution. Formulating the best solution(s) will necessitate creating a multidisciplinary team of clinicians (MD's, physiotherapists and other non-MD clinicians), health administrators and representatives of the public/patient population. Their first task would be to survey the existing data and map the need for further research, some of which are presented here. It is also likely that any proposed solutions will meet opposition from forces with special interests, a further justification for a multidisciplinary approach.

A PubMed review for orthopaedic medicine^m^ retrieved only 73 results, most of them not related to the topic of how orthopedic medicine is organized. There were a few relevant results from the 1980's (e.g. [55]) but they did not try to understand the source of the problem. An important treatise on the topic, aroused by the long waiting times in the UK for management of musculoskeletal disorders in the early 1990's discusses the lack of a specialty and complains that those orthopaedists practicing sports medicine are not actually trained in sports medicine [56]. Another problem mentioned is that non-orthopaedic surgeons practicing sports and musculoskeletal medicine have no specialist level training. The main suggestion is to train rheumatologists in musculoskeletal medicine, a possibility that doesn’t seem to have materialized in the UK, and is unlikely to become relevant in most countries including Israel.

The first and probably most important interventions should be in medical school. An issue as central as musculoskeletal pain must be dealt with in medical school when the thinking processes of the future doctors are moulded. The battle for teaching hours in medical school is a fierce one. Most programs have not seriously reviewed how some 10,000 hours of tuition might be best allocated to prepare doctors for the second quarter of the 21st century. Most curriculum decisions are highly political, with university accreditation based on hours of teaching medical students. It has long been known that the hospital inpatient medicine domination of the curricula impairs the preparation of students for the role of primary physicians, a role recommended to be strengthened by official commissions [1]. In the field of musculoskeletal medicine this problem is much more severe. Just as students are exposed to both cardiac surgery and cardiology, and spend (or certainly should spend) more time learning cardiology than cardiac surgery, so they should spend more time learning the diagnosis and management of medical orthopaedic ailments than they do the surgical problems of the musculoskeletal system. Even if this need is generally accepted, there are not enough teachers to teach orthopaedic medicine and a cadre of clinicians would need to be created. One possibility is multidisciplinary/combined clerkships as have been tried in some medical schools.

A second possibility is increasing the training of family medicine specialists. This option obviously makes a lot of sense, as they are at the front line of patient care and they therefore have a great influence on the management of the patient. They are also supposed to have a more comprehensive view of the patient. Indeed postgraduate training programs for primary physicians have been proven effective in some aspects of improving the interaction between primary care groups and orthopaedic outpatient clinics [57]. But while this is one of the more important directions, we are not sure whether this is realistic for a vast majority of family specialists, who are becoming more overworked with routine medical follow-up and various preventive medicine programs. This overload is causing a greater lack of family medicine specialists than the overall shortage of physicians the Israeli medical system is moving towards [58]. While those family specialists that have become interested in musculoskeletal medicine and managed to learn the clinical skills are definitely a major asset to the medical profession, this cannot substitute for a formal specialty, just as family specialists further specializing in infectious diseases or taking a special interest in hypertension or cardiology cannot obviate the need for these specialties. The field of orthopaedic medicine is too large. So while family specialists certainly need a better grasp of orthopaedic medicine, there is also a need for a specialty in the field they can consult or refer patients to, when appropriate.^n^

A third possibility is a residency in orthopaedic medicine. We propose that there is enough information to be learnt and clinical skills to be attained in orthopaedic medicine to fill a five year residency program. Beyond the classical training relating to surgical decision making, a specialist in orthopaedic medicine must also be proficient in assessing non-surgical complaints, including all those domains mentioned in Table 2 and Figure 3 and probably several others. The diagnosis and treatment of muscle complaints include innumerous subfields including myofascial release, stretching and massaging muscles, advising the patient (how and when, for a specific problem or after a specific sport activity) prescribing work & exercise limitations (temporarily or permanently) in case of injury or illness and prescribing orthotics and ergonomic solutions.

A fourth possibility is incorporating orthopaedic medicine into other medical specialties. Possible candidates are rehabilitation (physical medicine and rehabilitation, known in some countries as physiatry) and rheumatology. While some specialists in these fields have in-depth knowledge of musculoskeletal medicine, most do not. Most rehabilitation specialists in Israel take more interest in neurologic rehabilitation than in any aspect of physical medicine, and they do not seem to be accessible to the general public. The rheumatology specialization is usually undertaken after a four year specialization in internal medicine, and most specialists in rheumatology justifiably take interest in the highly specialized field of modulating the immune system. The relatively small numbers of MD's in these specialties limits the likelihood that a solution to the orthopaedic medicine lacuna will come from them (the Maccabi Healthcare Service does not seem to have even one independent rehabilitation specialist, and only 9 rheumatologists, Table 1).

A fifth possibility is adding training to orthopaedic surgeons. This could be during their internship or after they are board certified. One of the HMO's has considered making a compulsory course for newly contracted orthopaedic surgeons, but the planned course mainly targeted administrative regulations, and, as stated, the scope of orthopaedic medicine needs much more teaching and supervising time than possible in this sort of course.

A sixth possibility is making orthopaedic medicine a subspecialty. Subspecialties in orthopaedic surgery are a very hot topic, with most of the department chairmen against this trend of what is known as fragmentation of orthopaedic surgery. The advantages of specializing in a specific sub-field on surgical outcome are obvious and well known. The negative consequences of this fragmentation are that orthopaedic surgeons recently trained are only competent in a narrow area of orthopaedic surgery, and even less knowledgeable of general medical conditions that might be affecting their patient's complaint. Most orthopaedic chairmen are coming to realize that the proliferation of knowledge and techniques in each field makes it impossible to really encompass all subspecialties in a six year residency and really be a specialist in all these fields. De facto, as we previously stated, many residents decide in which subspecialty they want to work in before half their residency is over, and then spend more time in that field, sometimes even travelling abroad for a fellowship. As most residents in Israel do not end up working full time in hospital, and are therefore not working full time as surgeons, it might make sense that one of the options for the last two years of residency will be orthopaedic medicine, hitchhiking on the new trend for sub-specializing during the residency. According to this suggestion, they would get their certificate in orthopaedic surgery (pending passing the exams etc.) but also get a training and certificate in orthopaedic medicine. Issues that would still need to be resolved are who would teach the residents and where, and also who would replace the residents in their regular chores, the latter being the main reason so many orthopaedic surgeons that will inevitable not practice orthopaedic surgery are trained in Israel. A problem might be how attractive this subspecialty is likely to be to the personalities of those who choose orthopaedic residencies. This subspecialty should also be open to other specialties such as family medicine and rheumatology.

The seventh and last but not least option is to expand the role of non-MD's. One might hypothesize that most individuals that train in medicine for seven years do so because they are interested in the classical approaches (medication or surgery). This might lead to the conclusion that for diagnosing and treating the more mechanical parts of the body, it is necessary to train clinicians for this from very early on, paying much less attention to pharmaceutical and surgical treatments which are major components of traditional medical school curricula. This is what is done, to a certain extent by physiotherapists, osteopaths and chiropractors in many countries worldwide. Extending the scope of physiotherapists is part of the response in the UK to the long waiting lists for orthopaedists [59]. In Israel, where physiotherapy is the only one of these disciplines covered in national health insurance, the HMO's have not yet permitted direct access to these clinicians, even though this is permitted, and it is practiced in the private sector. The variation between the training programs for physiotherapists is enormous, and the concern whether their basic training enables them to recognize complaints relatable to serious disease (red flags) cannot be overlooked (but is also beyond this paper's scope). Two advantages in the idea of empowering physiotherapists are that this is the only health profession in which there is no shortage (and they even seem to be in excess), and that their basic training is shorter and less expensive. One of the major hindrances at present in Israel is the concern that patients will go from one discipline to another, raising expenditure. Another is the delay in the recognition of osteopathy by the Ministry of Health. So integrating these disciplines into the medical system is certainly going to need a lot of thought and balancing. It will also take many years before the public in Israel will give up going to their doctor when they have a problem. In summary, the potential for better utilization of these disciplines, in the light of the anticipated shortage of MD's in Israel must not be overlooked.

These directions are laid down as an opening for debate. As is customary in most settings, improving the service in musculoskeletal medicine while making it more efficient will probably be achieved by a combination of several of the directions mentioned. This should be decided on with a multidisciplinary approach, based on as much validated information as possible. But none of this is likely to take place until we acknowledge the need for the discipline of orthopaedic medicine and try to manage it effectively.

## Endnotes

^a^While this is a relatively well known fact, we are not aware of any reference on this.

^b^Physicians contracted and compensated per patient per calendar quarter.

^c^The second largest of Israel’s non-profit HMO’s serving approximately 1.8 million.

^d^Particularly as family specialists and pediatricians are considered primary care physicians in Israel. These data may be misleading, as they do not detail encounters nor patients, and they do not take working hours into account, but they are the only data officially available, and as orthopaedic encounters are short (only dermatology encounters are shorter) and the revisit rate is low, this data can give some idea. It may be supported by the fact that 3% of all medical encounters are with orthopaedist's, legend, Figure 1.

^e^There are two other important contributing factors; one is the reduction of working days a resident has resulting from regulations regarding time off after night shifts, and the other is the shortage of hospital posts, forcing the departments to send residents to work at community clinics. The latter is very problematic. While a resident works in the hospital outpatient clinic he is in training and under supervision. Working in a community clinic as a specialist, without the proper training or supervision, does not improve his skills, and is also deceptive of the public, to a certain extent.

^f^Neuro-linguistic programming (NLP) is an approach to communication, personal development, and psychotherapy.

^g^This was partly because the coverage in the Clalit became the basic coverage in the national insurance law.

^h^Albeit the British Journal of Bone and Joint Surgery has recently been renamed the Bone and Joint Journal, possibly as an acknowledgement of this problem.

^i^This is true in Israel. In some countries, orthopaedic trauma (such as a pelvic or femoral fractures) is treated by the traumatologist whose basic training is general surgery.

^j^Red flags are signs that should alert the clinician that this might not be a simple musculoskeletal complaint, i.e. that there might be something serious behind the complaint warranting a deeper investigation.

^k^Most adults are injected with 20–25 milliCurie of MDP-Tc^99m^, with a dose equivalent of 6–7 μSv. Data on the number of scans is based on how many generators are sold from the Soreq Nuclear Research Center. In 1998, this was about 60,000, when the population of Israel was about six million [45,46].

^l^("orthopaedic medicine" OR "orthopedic medicine") AND Eng[la].

^m^Another important role of orthopaedists is continuing medical education for non-orthopaedists. But meetings like this have frequently been initiated and failed, because two populations talk in different languages, the surgeons thinking of surgery for a surgically selected population, and the physicians needing other tools. That is exactly why there is a need for orthopaedic medicine. Furthermore, for newer trends in medicine such as expert patient programmes, where training of patient "specialists" for educating other patients has been shown effective in arthritis [52,53], the specialist in orthopaedic medicine is likely to be a better candidate for a multidisciplinary team than the surgeon.

^n^e.g. prolotherapy and mesotherapy.

^o^We prefer the term exercise medicine, which relates to any physical activity someone might be participating in such as walking or jogging, rather than sports medicine, which is sometimes limited to activities with a competitive nature. In as much, playing chess might be a sport, but it is not an exercise in our context.

## Competing interests

All authors declare they have no competing interests.

## Authors' contribution

ASF conceived the study, collected the data, drafted the manuscript and approved the final version. SV contributed to the conception of the study, revised the manuscript in the field of IMS and approved the final version. CM contributed to the conception of the study, revising the manuscript and approved the final version. AL contributed to the data acquisition, drafted the manuscript in the field of primary medicine and approved the final version. SM contributed to the conception of the study, revised the manuscript in the field of occupational medicine and approved the final version. GA contributed to the conception of the study, revised the manuscript in the field of orthopaedic surgery and training students and orthopaedic surgeons and approved the final version. DG contributed to the conception of the study, revised the manuscript in the field of health administration and approved the final version. All authors read and approved the final manuscript.

## Authors' information

ASF is an Israeli trained orthopaedic surgeon. He spent many years in the military, including heading the IDF orthopaedic service.

SV is a specialist in internal medicine and pain relief, head of the Rambam Institute of Pain Medicine. His main focus in pain is musculoskeletal pain. He established the Rambam School of Pain medicine to develop and teach postgraduate courses in pain medicine.

CM is a USA trained orthopaedic surgeon, professor of orthopaedics at the Hebrew University of Jerusalem. He has studied overuse injuries and set down the foundations for present day understanding of stress fracture biomechanics and patho-mechanism.

AL is chairman of the Department of Family Medicine at Hebrew University, Jerusalem, and of the Clalit Health Services, Jerusalem district. He currently chairs the Israeli National Committee for the Health of the Community. He also completed an MPH in general preventive medicine at the University of Washington School of Public Health in Seattle.

SM is head of the Jerusalem and Shefala district occupational medicine department in the Maccabi Health Services. He is currently the head of the resident training program in occupational medicine at the Tel Aviv University medical school.

GA is chairman of orthopaedic surgery at the Assaf HaRofeh Medical Center. His main fields of interest are knee and shoulder surgery, and training orthopaedic residents and MD students, for the latter he has won numerous awards. He has chaired the Israel Society for Knee Surgery and Arthroscopy, and has served as treasurer of the Israeli Orthopaedic Society for many years.

DG is an Associate Professor and Chairman of the Department of Health Systems Management at the Faculty of Health Sciences and the Guilford-Glaser Faculty of Business and Management at Ben-Gurion University of the Negev in Israel. He is also affiliated with the Center for the Evaluation of Value and Risk in Health (CEVR) at The Institute for Clinical Research and Health Policy Studies at Tufts Medical Center, Boston, MA, and is an adjunct faculty at the Tufts University School of Medicine.
